# Challenging of ECMO application in pediatric restrictive cardiomyopathy: case report of a novel *TNNI3* variant

**DOI:** 10.3389/fcvm.2024.1365209

**Published:** 2024-05-24

**Authors:** Yuxi Jin, Juan Xu, Yimin Hua, Haiyang Zhang, Yifei Li

**Affiliations:** Key Laboratory of Birth Defects and Related Diseases of Women and Children of MOE, Department of Pediatrics, West China Second University Hospital, Sichuan University, Chengdu, Sichuan, China

**Keywords:** restrictive cardiomyopathy, ECMO, *TNNI3*, heart failure, heart transplantation

## Abstract

**Background:**

Restrictive cardiomyopathy (RCM) represents a rare cardiovascular disorder stemming from filament-associated genes. Nonetheless, treating RCM presents considerable challenges, particularly concerning device implantation and mechanical support. Furthermore, elucidating the molecular function of specific variants holds promise in benefiting patients and enhancing prognosis, given the significant heterogeneity among RCM variants.

**Case presentation:**

The proband, an eight-year-old female, was admitted to our hospital post cardiopulmonary resuscitation due to sudden cardiac arrest. Echocardiography revealed bilateral atrial enlargement. Whole-exome sequencing uncovered a novel heterozygous mutation (c.509G>A, p.R170Q) in TNNI3. Evaluation using the MutationTaster application deemed c.509G>A pathogenic (probability = 0.99). Following clinical manifestations, imaging assessments, and genetic screening, the proband received an RCM diagnosis. ECMO was recommended along with continuous renal replacement therapy. However, persistent atrial flutter ensued post-ECMO withdrawal. Attempts to restore cardiac rhythm with cardioversion, metoprolol, and amiodarone proved futile. Subsequent heart failure led to the patient's demise due to cardiac shock. Based on crystal protein structural analysis, we observed that cTnI-R170Q and R170W exerted similar impacts on protein structural stability and formation. However, both differed significantly from cTnI-R170G, primarily influencing amino acid regions 32–79 and 129–149, involved in TnC and actin binding. Therefore, cTnI-R170Q was revealed to induce RCM via the same molecular mechanism as cTnI-R170W.

**Conclusion:**

Managing RCM remains a critical challenge. This study underscores the discouragement of device implantations for cardiac pump functional support in RCM, particularly for non-short-term scheduled HTx. Additionally, considering catheter ablation for atrial fibrosis-induced AFs is recommended. Mechanistically, cTnI-R170Q primarily diminishes troponin-actin interactions and destabilizes thin filaments.

## 1 Introduction

Restrictive cardiomyopathy (RCM) is a rare cardiovascular disorder linked to adverse clinical outcomes in the absence of heart transplantation (HTx) (1, 2). Typically affecting pediatric patients, RCM often leads to severe cardiac diastolic dysfunction, characterized by enlarged bilateral atria and restricted movement of the ventricular myocardium (1, 3).Therapeutic benefits from medication are markedly limited, prompting consideration of device implantation as an alternative for end-stage patients awaiting HTx (4). Studies have indicated that the survival rate of RCM patients on the HTx waiting list surpasses that of dilated cardiomyopathy, reaching up to 82% at the 1-year follow-up. However, device application has been identified as a risk factor for sudden cardiac death (SCD) and unfavorable prognoses (5). Recent reports explore the use of extracorporeal membrane oxygenation (ECMO), ventricular assist devices, and intra-aortic balloon pumps to bridge end-stage RCM patients to HTx. Yet, the prolonged duration of HTx waiting and the relatively acceptable device-free survival rate in RCM pose challenges to therapeutic strategies, particularly regarding ECMO support in children experiencing SCD after cardiopulmonary resuscitation.

RCM often manifests as an inherited disease within familial contexts (6–9). The genetic underpinnings of RCM encompass mutations or alterations in genes responsible for maintaining heart muscle structure and function. RCM can stem from both inherited and non-inherited causes, with genetic mutations playing a significant role. Several genetic disorders associated with RCM have demonstrated their pathogenicity. Mutations in genes encoding sarcomere proteins—such as *MYH7*, *MYBPC3*, *TNNT2*, *TNNI3*, and *TPM1*—have been linked to RCM (9–12). These genes play crucial roles in heart muscle fiber contraction and relaxation. Notably, *TNNI3* primarily implicates pediatric patients and stands out as one of the most specific genes associated with RCM. Furthermore, rare non-sarcomere variants—including *FLNC* and *Desmin*—have been identified in RCM, displaying significant heterogeneity that predominantly leads to DCM. Point mutations related to RCM have been pinpointed in *TNNI3*, primarily clustered within its C-terminal region (13–16). These RCM-related mutations often exhibit heightened Ca2+-sensitivity in the actin-myosin interaction. Research has highlighted modified interactions among sarcomeric proteins, particularly cMyBPC, which directly interacts not only with actin but also with troponin, significantly contributing to increased Ca2+-sensitivity at the sarcomere level (17). The presence of mutant troponin proteins induces impaired cTnI-tropomyosin interaction and inadequate decoration of the thin filament, leading to reduced dynamics. Intriguingly, a study by Cimiotti et al. emphasized variants at the same site, p.R170G and p.R170W, exhibiting stronger and weaker interactions between cTnI and tropomyosin, respectively. However, both variants indicated a similar level of enhanced Ca2+ sensitivity and disease phenotype, suggesting that different variants at the same site may entail controversial molecular mechanisms. Thus, exploring potential RCM variants and understanding their clinical implications remains crucial.

In this report, we present a case of RCM featuring a novel genetic variant, *TNNI3* c.509G>A p.R170Q. The patient received ECMO support following sudden cardiac arrest and subsequent heart failure. Despite ECMO withdrawal, persistent atrial flutter (AF) persisted, and multiple therapies proved ineffective in terminating AF. Tragically, the child succumbed two days after ECMO discontinuation. This case raises challenges concerning ECMO application in RCM treatment and the management of associated arrhythmias. Additionally, further investigation is warranted to determine the pathogenicity of this novel variant.

## 2 Methods

The study was approved by the ethics committee of the West China Second Hospital of Sichuan University (approval number 2021-069). In addition, we obtained written, informed consent from the patient's parents prior to performing WES and for the inclusion of the patient's clinical and imaging details in publications.

The genetic test had been performed at 8-years-old. The peripheral blood sample was obtained from the patient in an ethylenediaminetetraacetic acid (EDTA) anticoagulant blood sample tube that stored at 4°C for less than 6 h. DNA was extracted using the Blood Genome Column Medium Extraction Kit (Tiangen Biotech, Beijing, China) according to the manufacturer's instructions. WES was performed using the NovaSeq 6000 platform (Illumina, San Diego, CA, USA), and the raw data were processed using FastP to remove adapters and filter low-quality reads. Paired-end reads were aligned to the Ensembl GRCh37/hg19 reference genome using the Burrows–Wheeler Aligner. Variant annotation was performed in accordance with database-sourced minor allele frequencies (MAFs) and practical guidelines on pathogenicity issued by the American College of Medical Genetics. The annotation of MAFs was performed based on the 1000 Genomes, dbSNP, ESP, ExAC, and Chigene inhouse MAF database, Provean, Sift, Polypen2_hdiv, and Polypen2_hvar databases using R software (R Foundation for Statistical Computing, Vienna, Austria).

## 3 Case presentation

### 3.1 Clinical presentation and physical examination

The proband, an eight-year-old female, was admitted to our hospital following cardiopulmonary resuscitation due to sudden cardiac arrest. She reported a two-month history of reduced activity tolerance accompanied by bilateral pedal edema and periorbital edema. Additionally, she experienced fatigue, chest tightness, and shortness of breath after engaging in physical activity, typically lasting for 15 min. Over the month leading to hospital admission, her activity tolerance progressively declined. Notably, cyanosis of the complexion and lips became evident after mild activities. Approximately forty minutes before admission, the patient experienced a cardiac arrest during exercise. Emergency medical services arrived within six minutes and initiated cardiopulmonary resuscitation, administering two intravenous bolus injections of epinephrine. Spontaneous heartbeat and breathing resumed within ten minutes; however, the patient remained unconscious, experiencing recurrent ventricular fibrillation. Immediate asynchronous defibrillation was employed alongside tracheal intubation and continuous positive pressure ventilation using a manually operated resuscitation balloon. Subsequently, the patient was transferred to our intensive care unit (ICU).

Upon admission to the ICU, the initial physical examination revealed an acute critical illness characterized by severe diaphoresis, dizziness, and fatigue. The patient appeared pale and restless. The sinus rhythm heart rate was approximately 105 beats/min, while her blood pressure stood at 83/52 mmHg, maintained through continuous intravenous dopamine infusion. Notably, the patient exhibited absent orbital pressure and cough reflexes. Her nutritional status appeared normal, and she displayed no response to external stimulation. No surface trauma injuries were observed. Under mechanical ventilation, symmetrical respiratory movements were noted in both lungs, with rough breath sounds and occasional wheezing, yet no significant bilateral wet rales were detected. Cardiac examination revealed the apex of the heartbeat shifted to the lower left, accompanied by a sense of lift in the precordial area. A mildly enlarged heart boundary was observed, along with a heterogeneous rhythm characterized by premature beats. However, the heart sounds were notably dull. Abdominal examination revealed softness, with the liver palpable 2 cm below the subcostal margin and 1–2 cm below the xiphoid process, displaying a medium texture. The spleen was not palpable below the subcostal margin. Muscle strength and tension in all four extremities appeared normal. No pathological signs or signs of meningeal irritation were detected. The extremities were cold, presenting mottled skin, with a capillary refill time of 5 s.

Additionally, the patient's parents denied any positive family history of cardiac attacks, cardiovascular issues, hypertension, or coronary artery diseases. There was also no reported history of diabetes or obesity among family members. No inherited diseases, including cardiomyopathies or metabolic disorders, were identified in the family history.

### 3.2 Laboratory and imaging evaluation

**Figure 1:**
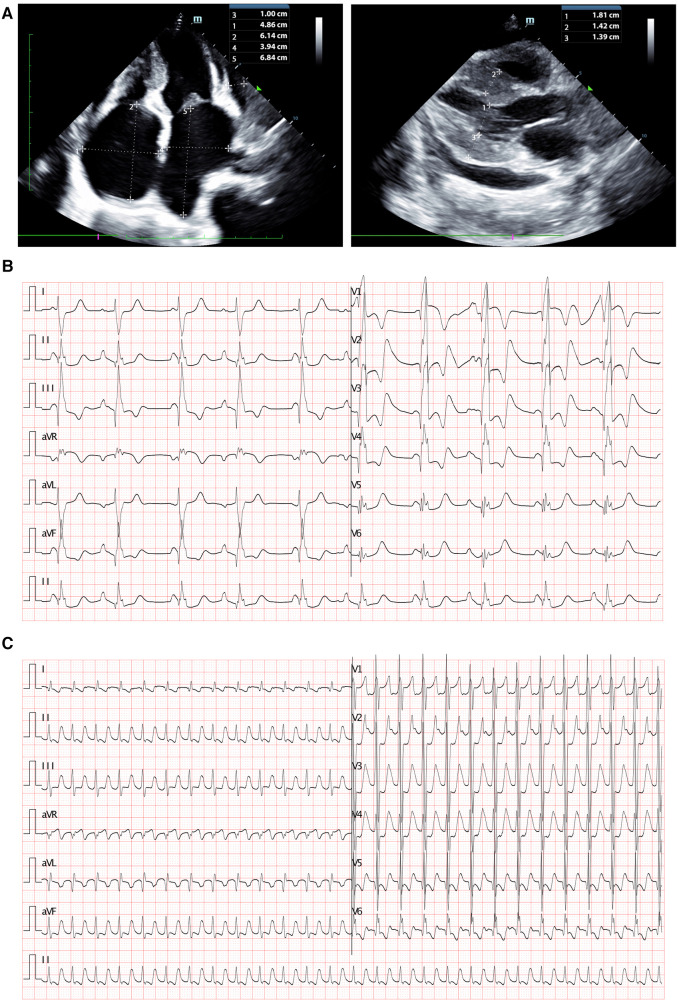
Clinical imagines of the proband. (**A**) Echocardiography demonstrated extremely enlarged bilateral atrium (left atrium dimeter 3.94 × 6.84 cm, right atrium dimeter 4.86 × 6.14 cm), interventricular septum segmentally thickened, partial ventricular wall pulse amplitude segmentally diffusely reduced and uncoordinated, mild mitral regurgitation, reduced left ventricular systolic and diastolic function. (**B**) Electrocardiogram revealed abnormal bilateral atrial signals, premature atrial beat, junctional ectopic beat, first degree atrioventricular block, complete right bundle branch block, prolonged QT interval, and abnormal *Q* wave. (**C**) Electrocardiogram presented persistent AF had been identified after ECMO withdraw.

Blood gas analysis revealed severe acidosis (pH 6.93, lactate > 15.0 mmol/L, HCO3- < −50 mmol/L, BE < −30) accompanied by carbon dioxide retention (PCO2 106 mmHg, PO2 98 mmHg). Blood cell counts and renal function showed no significant abnormalities. Hepatic function tests indicated mild liver injury (AST 119.8 U/L, n.v. < 42 U/L). Elevated blood sugar levels were noted at 23.7 mmol/L. Coagulation function tests displayed dysfunction (PT 16.94 s, APTT 64.58 s, D-dimer 11.2 ug/ml, FDP 37.83 s, INR 1.42). Furthermore, myocardial injury biomarkers were significantly elevated (cTnI 0.377 ng/ml, n.v. < 0.023 ng/ml; BNP > 5,000 pg/ml, n.v. < 5 pg/ml). Infectious parameters and viral antibodies returned negative results. Echocardiography revealed an extremely enlarged bilateral atrium (left atrium diameter 3.94 × 6.84 cm, right atrium diameter 4.86 × 6.14 cm), segmental thickening of the interventricular septum, segmentally diffuse reduction and lack of coordination in ventricular wall pulse amplitude, mild mitral regurgitation, and diminished left ventricular systolic and diastolic function identified by increased parameters of early diastolic velocity (*E* wave) and low late filling velocity (*A* wave) (Figure 1A). Chest CT indicated localized infection sites. Electrocardiogram findings displayed abnormal bilateral atrial signals, premature atrial beats, junctional ectopic beats, first-degree atrioventricular block, complete right bundle branch block, prolonged QT interval, and abnormal *Q* wave (Figure 1B). Subsequent to ECMO withdrawal during hospitalization, persistent atrial fibrillation (AF) was observed (Figure 1C).

### 3.3 Molecular results

**Figure 2:**
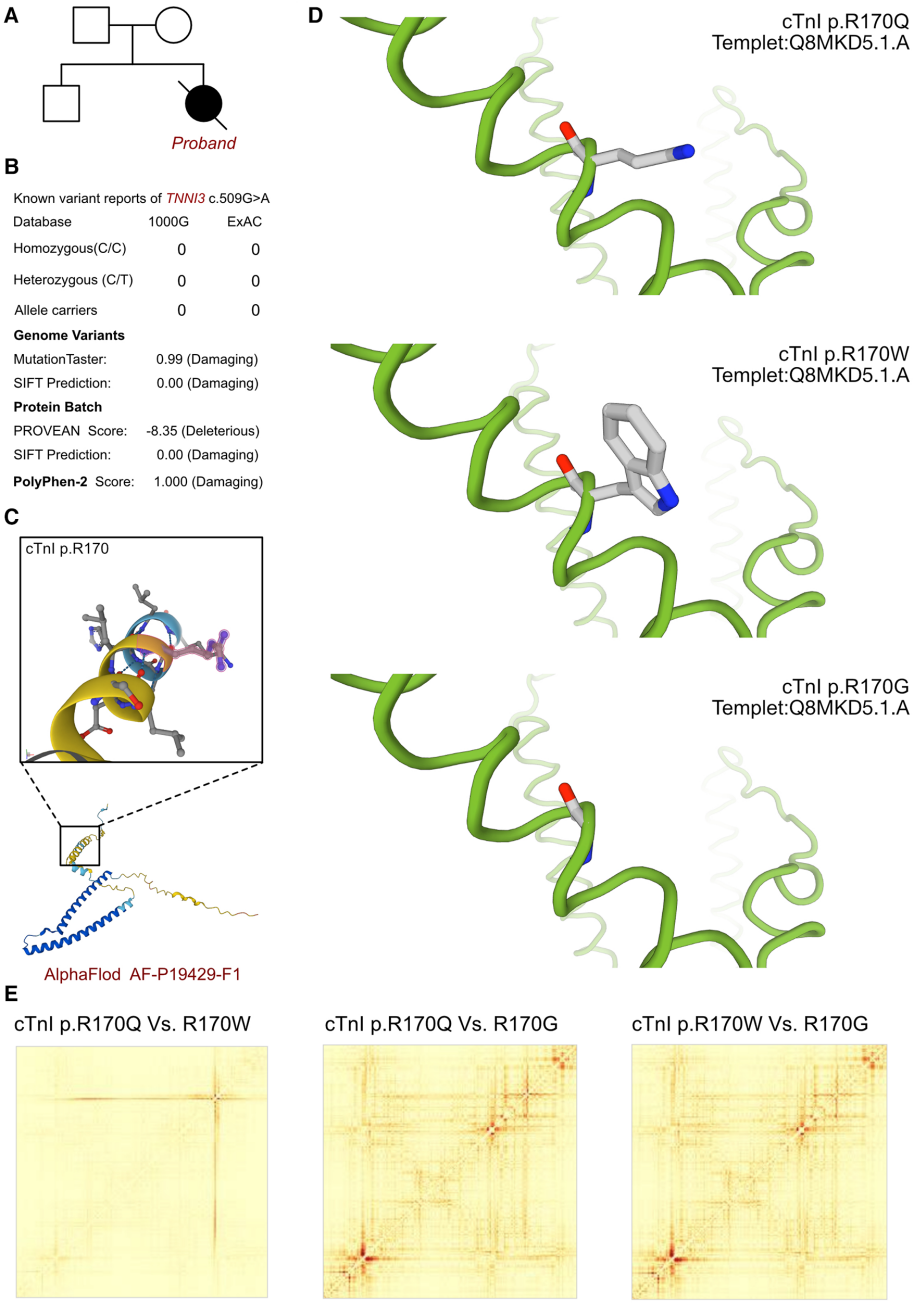
The *TNNI3* molecular analysis. (**A**) The proband exhibited a *de novo* heterozygous variant of *TNNI3* (c.509G>A, p.R170Q). (**B**) The variant of TNNI3 c.509G>A had never reported in 1000G and ExAC, it has predicted protein damaging by PolyPhen-2 and SFIT. (**C**) The protein structure of cTnI has been built and named AF-P19429-F1. (**D**) Localized structural views had been established by SWISS-MODEL for cTnI-R170Q, R170W and R170G based on Q8MKD5.1.A template, respectively. (**E**) Comparisons had been made on the structural impaired among cTnI-R170Q, R170W and R170G. Based on the ensemble variance analysis, we found that the cTnI-R170Q and R170W shared the similar impacts on protein structural stability and formation. However, both of them presented significant differences with cTnI-R170G, mainly influencing the regions of 32-79 and 129-149 amino acids, which were involved in TnC and actin binding.

As cardiomyopathy had been suspected, genetic test was involved to explore any associated variants. WES had been performed for this proband and her parents. According to the analysis result of WES, a *de novo* heterozygous variant had been identified as c.509G>A (p.R170Q) of *TNNI3* gene (Figure 2A). Such variant was not inherited from maternal or paternal side. And the variant of *TNNI3* c.509G>A was never reported in database which was considered as a novel variant (Figure 2B). Besides, we had excluded all the potential variants involved in cardiovascular and muscular disordered. Then we reviewed all the other variants which were reported as pathogenic or likely pathogenic ones, and none of them was confirmed to be associated with the phenotype of the proband. So that, we suspected the novel heterozygous variant of *TNNI3* c.509G>A contributed to the pathogenic phenotype of this proband as an RCM. To elucidate the molecular architecture of the human *TNNI3* gene, we used MutationTaster with R software to predict the pathogenicity of *TNNI3* c.509G>A (p.R170Q), and assess the impact of these mutations on protein structure. As there was no available full-length protein crystal structure for cTnI (encoded by *TNNI3*) which had been analyzed by x-ray or cryo-EM, AlphaFold protein structure software (https://alphafold.ebi.ac.uk/) tool had been used to predicted protein crystal structure. The protein structure of cTnI has been built and named AF-P19429-F1 (Figure 2C) (18, 19). Within the structure, two important domains had been revealed with analyzed crystal structure. The region of 32–79 amino acids was involved in troponin C (TnC) binding, and the region of 129–149 amino acids was involved in TnC and actin binding. Then we performed modeling analysis using the SWISS-MODEL (https://swissmodel.expasy.org/) for the mutant site in wild type with the AF-P19429-F1.A based Q8MKD5.1.A template. We estimated the capability of the protein structure using Ramachandran plots. According to the American College of Medical Genetics, the mutation c.509G>A has certain pathogenicity (PS4 + PM1 + PM2_Supporting + PM5 + PP3_Moderate + PS2). The analyses from MutationTaster revealed the variant of c.509G>A impaired the transcription of *TNNI3* leading to amino acid sequence and histone methylation site changes, was predicted as disease causing. As the amino acid 170 site had been found as a hot mutant site of *TNNI3*, the most reported variants were cTnI-R170W and R170G. Localized structural views had been established by SWISS-MODEL for cTnI-R170Q, R170W and R170G based on Q8MKD5.1.A template, respectively (Figure 2D). Previous research demonstrated that the amino acid 170 site mutation presented two different molecular mechanisms. cTnI-R170G binds stronger to actin than wildtype cardiac troponin, the dissociation constant K_D_ was significantly reduced by 40%. While cTnI-R170W showed a significantly increased K_D_ indicating a reduced binding to actin. And troponin containing cTnI-R170W integrated less sufficiently into the thin filament, leading to 50% filament breaks decreased for R170G and 25% for R170W. However, both of the mechanisms resulted in elevated Ca2+-sensitivity of contraction which mediated by cMyBPC at different level. Then we compared the structural impaired among cTnI-R170Q, R170W and R170G (Figure 2E). Based on the ensemble variance analysis, we found that the cTnI-R170Q and R170W shared the similar impacts on protein structural stability and formation. However, both of them presented significant differences with cTnI-R170G, mainly influencing the regions of 32–79 and 129–149 amino acids, which were involved in TnC and actin binding. So that, the cTnI-R170Q had been revealed as the same molecular mechanism in inducing RCM with cTnI-R170W, indicating a less sufficiently integrated in filament and higher filament breaks with less cMyBPC regulation.

### 3.4 Final diagnosis and treatment

Following an analysis of clinical manifestations, imaging assessments, and genetic screening, the patient was diagnosed with restrictive cardiomyopathy (RCM) harboring a novel *de novo TNNI3* variant. Given the patient's critical condition, ECMO was recommended along with continuous renal replacement therapy. Subsequently, positive supportive therapy led to the stabilization of cardiac function and circulation, prompting ECMO withdrawal on the tenth day. However, shortly after ECMO removal, persistent atrial fibrillation (AF) ensued. Attempts to restore normal cardiac rhythm through cardioversion, metoprolol, and amiodarone proved ineffective. Subsequent heart failure ensued, culminating in the patient's demise due to cardiac shock.

## 3 Discussion

RCM constitutes a rare cardiovascular disorder, accounting for 5% of diagnosed cardiomyopathies (20). RCM is characterized by heightened myocardial stiffness resulting in restrictive diastolic dysfunction. Molecular alterations in RCM often prompt biventricular myocardial interstitial fibrosis, leading to restricted ventricular end-diastolic volume. Among pediatric cardiomyopathies, RCM stands out as one of the most deteriorating disorders, with a two-year survival rate estimated below 50% (6). Additionally, pediatric RCM cases exhibit significant heterogeneity, contributing to diverse clinical presentations. However, end-stage RCM patients typically manifest symptoms associated with left heart failure, including dyspnea, hemoptysis, pulmonary edema, reduced cardiac output, thromboembolism, and even sudden cardiac death.

Therapeutic strategies for RCM remain under debate (21–24). Over the past decade, the rapid advancements in next-generation sequencing technology have significantly contributed to genetic testing and molecular diagnosis, playing a crucial role in managing inherited diseases. Oral medication for myocardial remodeling has shown limited benefits, while catheter assessment and device implantation are being considered for RCM patients. Diagnosis of RCM in some patients occurs through young-age cardiac screening or incidental findings, whereas others experience sudden cardiac arrest as the initial manifestation (2). Generally, HTx is regarded as the final resort and most effective approach for managing RCM. However, global variations in HTx waiting times necessitate vigilance regarding inevitable worsening of heart failure during follow-ups. Prior to HTx, bridging therapies have been introduced for some awaiting patients, including heart assist devices, ECMO, and intra-aortic balloon pumps. Notably, while device implementation has improved outcomes in dilated cardiomyopathy, RCM patients receiving such mechanical support have exhibited significantly increased mortality rates within a year (5). This suggests that myocardial pump function intervention may not yield adequate clinical outcomes. Pathological changes in RCM, along with treatment responses, remain primarily associated with fibrosis and functional impairment of the thin filament. ECMO played an exceptional role in rescuing the patient's life post-cardiac arrest by providing cardiac pump function support (25). However, severe complications such as non-reversible arrhythmia and aggressive diastolic impairment nearly led to fatality before HTx. Consequently, preserving systolic function warrants serious consideration. Previous reports suggested patients on ECMO was mostly applied in the patients in prior of Heart transplantation, serving as a bridge treatment. Generally, the prognosis of RCM with ECMO application was less satisfied than that of DCM. One reasonable indication for this patient to receive ECMO was the highest comorbidity during her treatment. As conventional support had been failed to provide efficient life support, and death was thought to be unavoidable without mechanical support application. Thus, ECMO was applied to this patient finally (26). Thus, we advocate that physicians should aim to minimize short-term device support, ensuring that mechanical therapies are closely aligned with HTx as a bridging strategy.

Lethal arrhythmias commonly coincide with the end-stage progression of RCM (21). Persistent high-pressure loading on the atria contributes significantly to atrial myocardial fibrosis and increased stiffness. RCM decreased ventricular diastolic function, which lead to bi-atrial enlargement and left ventricular stiffness, contributing to onset of arrhythmias. Additionally, the application of ECMO might affect the remodeling of atrium and cause a complex invasion between myofibroblasts and cardiomyocytes. Thus, the termination of ECMO may trigger a shear stress or hydrostatic pressure elevation, which would cause unstable calcium handling associated with atrial arrhythmia. Beyond this report, a few other reports also demonstrated the application of ECMO in RCM was associated with lethal arrhythmia, which brought great challenges in deciding ECMO treatment in RCM. Consequently, non-reversible AFs are frequently observed in RCM (8). Such AFs typically arise due to atrial reentry caused by extensive scarring in multiple areas. Anti-arrhythmic medications often prove ineffective in managing this emergent issue (4). Therefore, catheter ablation should be considered as an alternative for managing persistent AFs. Moreover, certain newly developed drugs have been investigated for their role in RCM treatment. Troponin-targeting agents like levosimendan and green tea extract (-)-epigallocatechin-3-gallate (EGCG) have demonstrated the ability to stabilize the structural integrity of reconstituted thin filaments and improve contractile function *in vitro* among specific TNNI3 patients, such as those with cTnI-D127Y mutations (26). However, not all *TNNI3* variants respond similarly to the mentioned therapies. Presently, none of these therapies directly address the underlying sarcomeric dysfunction associated with thin-filament mutations. As mounting evidence indicates that thin filament cardiomyopathies operate through distinct mechanisms, there is a pressing need for therapies tailored to address the unique underlying mechanisms specific to each patient, contingent upon their respective mutation.

Genetic analysis and molecular exploration remain pivotal fields in RCM research. Understanding the molecular alterations at each variant site is crucial in managing RCM patients. *TNNI3* stands as the most prevalent causal mutation for RCM, also linked to dilated and hypertrophic cardiomyopathies. In recent studies, 38%–50% patients with RCM identified pathogenic or likely-pathogenic variants, and 8%–28% patients were inherited with TNNI3 variants. But as the prevalence of RCM is extremely low, the proportion of TNNI3 in RCM varied a lot among reported studies. And more researches were required to demonstrate a convince result. Although the precious data of TNNI3 was missing, but the TNNI3 had been considered as the most common identified mutations in children RCM cases, indicating the TNNI3 variants would more likely to induce early onset RCM (26). Reported cases highlight that C-terminal variants, primarily in exon 7 and exon 8, induce RCM, whereas N-terminal variants are predominantly associated with other cardiomyopathies (16). A summary of all reported variants can be found in Table 1. Early clinical and molecular diagnosis of RCM hold significant importance. Ishida et al. reported that 50% of pediatric RCM patients exhibited pathogenic or likely-pathogenic gene variants, with *TNNI3* missense variants being the most frequent. Patients with pathogenic variants showed significantly lower transplant-free survival rates than those without pathogenic variants (2). Early diagnosis is critical for the survival of affected patients; those diagnosed through school screenings exhibited better transplant-free survival rates compared to those diagnosed due to heart failure symptoms (*P* = 0.0027). However, no significant differences were found in the ratio of patients diagnosed in nationwide school heart disease screening programs between positive and negative pathogenic variants. Consequently, 2- and 5-year survival rates were notably lower in patients with pathogenic variants (50% and 22%) compared to those without (62% and 54%; *P* = 0.0496), highlighting the crucial role of early RCM diagnosis and integrative management in improving prognosis. Furthermore, diverse variants exhibited heterogeneous medication responses, suggesting controversial molecular bases underlying various mutation sites. The proband demonstrated a novel variant, cTnI-R170Q, not previously reported. Understanding the molecular mechanism of R170Q in inducing RCM is crucial. A previous study revealed that the cTnI-R170 variant might alter two types of sub-cellular structures. Cimiotti et al. observed that filaments containing R170G/W appeared wavy and exhibited breaks (16). Filament decoration with myosin subfragment S1 was normal with R170W but irregular with R170G. Both R170W and R170G enhanced the interaction between troponin and tropomyosin, affecting actin binding either by strengthening (R170G) or weakening (R170W). Only cTnI-R170G showed reduced affinity towards cMyBPC-C0C2 linking to cTnC (17). Thus, RCM cTnI variants R170G/W impede communication between thin and thick filament proteins, destabilizing thin filaments. Comparing protein structural changes between R170W and R170G, our analysis indicated that R170Q and R170W exhibit similar impacts at the protein level structure. Both significantly differ from R170G, particularly in two domains (TnC binding and actin binding), aligning with findings from Cimiotti et al. R170Q reduces troponin-actin interaction while maintaining stability in binding to cMyBPC linked to TnC. Additionally, Ca2+-dependent hyper-activation of cardiac thin filaments and destabilization of thin filaments were observed in R170Q.

**Table 1:** Summarization of reported TNNI3 mutations resulting in RCM.

Reference.	Mutation site	Protein	Variant type	Onset age/gender	Diagnosis	Echo	ECG	CMR	Chest x-ray/ computed tomography	Clinical outcome
Mogensen J, et al. J Clin Invest. 2003	c.92C→A	Arg192His	Missense mutation	19 years/M	RCM	Bi-atrial enlargement	Prominent *P*-waves in all leads consistent with bi-atrial enlargement			Death
	c.87A→G	p.Asp190His	Missense mutation/M	11 years/M	RCM	Bi-atrial enlargement accompanied by a restrictive filling pattern	Bi-atrial enlargement			Alive
	c.886A→G	p.Lys178Glu	Missense mutation	6 years/F	RCM		Bi-atrial enlargement			Alive
	c.799C→T n	p.Arg145Trp	Missense mutation	68 years/F	RCM		Bi-atrial enlargement, ST-segment changes			
	c.799C→T n	p.Arg145Trp	Missense mutation	70 years/M	RCM		Atrial fibrillation, left ventricle hypertrophy, ST-segment changes			
	c.856G→A	p.Ala171Thr	Missense mutation	63 years/M	RCM		Atrial flutter, right bundle branch block, ST-segment changes			
	c.797T→A	p.Leu144Gln	Missense mutation	31 years/F	RCM		Sinus rhythm, bi-atrial enlargement, ST-segment abnormalities, *Q* waves			Death
Kaski JP, et al. Heart. 2008	g.4792A→G	p.Lys144Glu	Missense mutation	7 years/F	RCM		Sinus rhythm, bi-atrial enlargement, ST-segment abnormalities			
	g.4789_4790delAA	Glu177fsX209		6.4 years/F	RCM		Atrial fibrillation, ST-segment abnormalities			
Kostareva A, et al. Int J Cardiol. 2009	Nt4762delG		Frame shift	23 years/F	RCM	Remodeling of the left ventricle with decreased left ventricular mass index				Death
Yang SW et al., Cardiol Young. 2010		p.Arg240His	Missense mutation	18 months		Left atrial enlargement		Left atrial enlargement and thickening of the atrial walls, dilation of the pulmonary artery and veins		
van den Wijngaard A et al., Neth Heart J. 2011	c.434G>A	p.Arg145Gln	Missense mutation	<9 years/F	RCM					
	c.549+2delT	not determined		<1 years/F	RCM					Death
	c.532_534delAAG	p.Lys178del	Missense mutation	<11 years?/M	RCM					
	c.433C>T	p.Arg145Trp	Missense mutation	19 years/M	RCM?					
Yang SW, et al. Zhonghua Xin Xue Guan Bing Za Zhi. 2013	c.611G>A	p.Arg204His	Missense mutation	8 years/F	RCM and ventricular septal defect	Left atrial enlargement, restricted flow spectrum, and impaired left ventricular diastolic function	*P* wave change, ST segment descends of lower wall lead			Alive
	c.575G>A	p.Arg192His	Missense mutation	10 years/F	RCM	Bi-atrial enlargement, restricted flow spectrum, and impaired left ventricular diastolic function	Sinus rhythm, biatrial enlargement, extensive *T*-wave changes			Alive
Chen Y, et al. J Biomed Res. 2014	c.575G>A	p.Arg192His	Missense mutation	12 years	RCM	Biatrial enlargement, left ventricular diastolic dysfunction	Sinus rhythm, biatrial enlargement, diffuse *T*-wave changes, mild short PR interval	No delayed gadolinium enhancement and enlargement in both atria		
Mouton JM et al., Cardiovasc J Afr. 2015	c.432TG>AT	p.Leu144His	Missense mutation	27 years/F	RCM	Bi-atrial dilatation	P-mitrale and partial right bundle branch block		Pulmonary congestion	Death
Ruan YP, et al. Chin Med Sci J. 2016	c.448A→G	p.Ser150Pro	Missense mutation	51 years/M	RCM	Myocardial involvement disorder, biatrial enlargement, mild mitral valve regurgitation, mild and modest tricuspid valve regurgitation, reduced systolic and diastolic function of left ventricle and pericardial effusion				
Ding WH, et al. Chin Med J (Engl). 2017		p.Arg192His	Missense mutation	between 5 and 12 years	RCM					
		p.Arg204His	Missense mutation	between 5 and 12 years/M and F	RCM					
Hwang JW, et al. Korean Circ J. 2017	c.433C>T	p.Arg145Trp	Missense mutation	52 years/F	RCM	Bi-atrial enlargement and a restrictive filling pattern	Atrial fibrillation and ST-T abnormalities in inferolateral leads	Mid and basal wall myocardial fibrosis	Interval-increased extent of markedly dilated both atria, with thrombi in the right atrium	Death
Pantou MP, et al. BMC Med Genet. 2019	c.586G>C	p.(Asp196His)	Missense mutation	41 years/F	RCM	Borderline contractile function, biatrial dilatation and mild mitral regurgitation	Atrial fibrillation			Alive
Cimiotti D, et al. PLoS One. 2020	c.508C>G	p.Arg170Gly	Missense mutation	3 years/M	RCM					Death
	c.508C>T	p.Arg170Trp	Missense mutation	8 years/13 months	RCM	Restrictive filling pattern and enlarged atria				Death
Ueno M, et al. J Cardiol Cases. 2020	c.574C>T	p.Arg192Cys	Missense mutation	11 years/F	RCM and ventricular septal defect	Dilation of both atria, decreased diastolic function	Bi-lateral atrial and LV hypertrophy pattern, and ST-T-wave abnormalities	Both atrial dilation, and hypertrophy of the septal and inferior LV wall, LGE was positive	Cardiomegaly, left atrial dilation, and dull costophrenic angles	Death
				11 years/F	RCM		Abnormal *P*-waves in all leads			
Jia L, et al. Zhonghua Yi Xue Yi Chuan Xue Za Zhi. 2021	c.549+1G>T	not determined	deletion variation	1 year and 6 months/M	RCM	Pericardial effusion, patent foramen ovale, bi-atrial enlargement, widening of pulmonary artery, mild regurgitation of mitral and tricuspid valves	Sinus rhythm, right atrial hypertrophy, ST-T changes			
	c.433C>T	p.Arg145Trp	Missense mutation	4 years and 1 month/M	RCM	Bilateral atrial enlargement, biventricular stenosis, local thickening of left ventricular wall, mild tricuspid valve regurgitation, pericardial effusion	Sinus rhythm, *P*-wave, ST-T segment changes			
Ji L, et al. Front Cardiovasc Med. 2022	c.611(exon8)G>A	p.Arg204His	Missense mutation	7 years/M	RCM	Bi-atrial enlargement, decreased diastolic function, moderate mitral valve regurgitation	Ptf-V1<–0.04 mms and biphasic *P* waves	No abnormalities	Enlarged heart shadow	Alive
Zheng M, et al. BMC Cardiovasc Disord. 2022	c.517C>T	p.Leu173Phe	Missense mutation	4 months/M	RCM	Bi-atrial dilation, decreased left ventricular ejection fraction, impaired left ventricular diastolic function	Bi-atrial enlargement, diffuse ST-T wave changes.			Death
	c.508C>T	p.Arg170Trp	Missense mutation	4 yearsand 5 months/F	RCM	Bi-atrial and right ventricle enlargement, decreased left ventricular ejection fraction, impaired left ventricular diastolic function	Bi-atrial enlargement and diffuse ST-T wave changes.	Bi-atrial enlargement, thickened pericardium	Increased heart shadow	Death
	c.575G>A	p.Arg192His	Missense mutation	5 years/F	RCM	Dilation of bi-atria, decreased left ventricular ejection fraction, impaired left ventricular diastolic function	ST-T wave changes	Bi-atrial enlargement	Increased heart shadow	Death
Gerhardt T, et al. Eur Heart J Case Rep. 2022		p.Lys193Glu		27 years/F	RCM	Bi-atrial dilatation, ompromised diastolic function with a restrictive filling pattern and pericardial effusion	Atrial fibrillation	Gross atrial dilatation		Alive
Dai HL, et al. J Int Med Res. 2023	c.574C>T	p.Arg192Cys	Missense mutation	8 years/F	RCM	Biatrial enlargement, pericardial effusion, impaired left ventricular diastolic function	Biatrial enlargement, ST-segment depression in the inferior and lateral precordial leads	Biatrial enlargement and late gadolinium enhancement was negative.	Normal coronary artery, normal	Death
Fangjie Wang et al. Zhonghua Yi Xue Yi Chuan Xue Za Zhi. 2023.	c.508C>T	p.Arg170Trp	Missense mutation	2 years and 4 months/M	RCM and phenylketonuria	Bi-atria and the right ventricle enlargement, impaired ventricular diastolic function, pericardial effusion	Sinus bradycardia, *P* wave change, prolonged PR interval			
Hasegawa M, et al. Dev Growth Differ. 2024	c.508C>T	p.Arg170Trp	Missense mutation	2 years	RCM	Dilated left atrium				

Echo, echoocardiography; ECG, electrocardiography; CMR, cardiac magnetic resonance; RCM, restrictive cardiomyopathy; LGE, late gadolinium enhancement.

## 4 Conclusions

In summary, we presented a rare case of RCM involving a novel *TNNI3* c.509G>A variant. However, managing RCM remains a critical challenge, with HTx standing as the most efficient approach for end-stage patients. Early diagnosis of *TNNI3*-associated RCM significantly improves prognosis and allows ample time for HTx preparation. Device implantation for cardiac pump support in RCM is not recommended for patients not imminently scheduled for HTx, suggesting that such mechanical interventions should serve as tightly bridged therapies preceding HTx. Atrial fibrosis can lead to persistent AF, necessitating consideration for catheter ablation. Furthermore, the molecular mechanism of R170Q was elucidated through comparative crystal protein structural reconstruction, revealing that R170Q primarily diminishes the interaction between troponin and actin, destabilizing thin filaments.

## Data Availability

The original contributions presented in the study are publicly available. This data can be found here: https://www.ncbi.nlm.nih.gov/bioproject/1109441.
